# Exploring the Diversity of *Gardnerella vaginalis* in the Genitourinary Tract Microbiota of Monogamous Couples Through Subtle Nucleotide Variation

**DOI:** 10.1371/journal.pone.0026732

**Published:** 2011-10-25

**Authors:** A. Murat Eren, Marcela Zozaya, Christopher M. Taylor, Scot E. Dowd, David H. Martin, Michael J. Ferris

**Affiliations:** 1 Department of Computer Science, University of New Orleans, New Orleans, Louisiana, United States of America; 2 Josephine Bay Paul Center for Comparative Molecular Biology and Evolution, Marine Biological Laboratory, Woods Hole, Massachusetts, United States of America; 3 The Research Institute for Children, Children's Hospital, New Orleans, Louisiana, United States of America; 4 Research and Testing Laboratory, Lubbock, Texas, United States of America; 5 Section of Infectious Diseases, Louisiana State University Health Sciences Center, New Orleans, Louisiana, United States of America; 6 Departments of Pediatrics and Microbiology Immunology and Parasitology, Louisiana State University Health Sciences Center, New Orleans, Louisiana, United States of America; Institute for Genome Sciences, University of Maryland School of Medicine, United States of America

## Abstract

**Background:**

Bacterial vaginosis (BV) is an enigmatic disease of unknown origin that affects a large percentage of women. The vaginal microbiota of women with BV is associated with serious sequelae, including abnormal pregnancies. The etiology of BV is not fully understood, however, it has been suggested that it is transmissible, and that *G. vaginalis* may be an etiological agent. Studies using enzymatic assays to define *G. vaginalis* biotypes, as well as more recent genomic comparisons of G. *vaginalis* isolates from symptomatic and asymptomatic women, suggest that particular *G. vaginalis* strains may play a key role in the pathogenesis of BV.

**Methodology/Principal Findings:**

To explore *G. vaginalis* diversity, distribution and sexual transmission, we developed a Shannon entropy-based method to analyze low-level sequence variation in 65,710 *G. vaginalis* 16S rRNA gene segments that were PCR-amplified from vaginal samples of 53 monogamous women and from urethral and penile skin samples of their male partners. We observed a high degree of low-level diversity among *G. vaginalis* sequences with a total of 46 unique sequence variants (oligotypes), and also found strong correlations of these oligotypes between sexual partners. Even though Gram stain-defined normal and some Gram stain-defined intermediate oligotype profiles clustered together in UniFrac analysis, no single *G. vaginalis* oligotype was found to be specific to BV or normal vaginal samples.

**Conclusions:**

This study describes a novel method for investigating *G. vaginalis* diversity at a low level of taxonomic discrimination. The findings support cultivation-based studies that indicate sexual partners harbor the same strains of *G. vaginalis*. This study also highlights the fact that a few, reproducible nucleotide variations within the 16S rRNA gene can reveal clinical or epidemiological associations that would be missed by genus-level or species-level categorization of 16S rRNA data.

## Introduction

As a group, bacteria are the most genetically diverse and abundant life form on Earth [1]. In fact the human body is home to a diverse assemblage of bacteria that colonize the gastrointestinal tract, oral cavity, skin, airway passages and genitourinary system [2]. Culture-independent surveys estimate that the human gut alone is home to 40,000 bacterial species [3] and it is estimated that the number of bacterial cells in the human body is ten-fold greater than the number of eukaryotic cells that comprise the human body [4], [5]. Humans depend on a symbiotic relationship with bacteria to extract nutrients from food and for normal immune system development [6]–[8]. On the other hand, adverse medical conditions are also associated with changes in the composition and relative abundance of our bacterial microbiota.

One of the most well studied medical conditions associated with a change in the human microbiota is bacterial vaginosis (BV). BV is a common vaginal disorder and symptoms often include vaginal discharge, pruritis, and odor. The microbiology of BV is characterized by a drastic reduction in the concentration of *Lactobacillus* species in the vaginal environment and an increase in the concentration of *G. vaginalis* and many other bacterial genera [9]. This shift in microbiota is reflected in quantifiable changes in vaginal smear Gram stains (GS) as measured by the Nugent Score (NS) [10]. Women with *Lactobacillus* dominated microbiota have NS of 0–3 while women with BV have NS of 7–10. It is important to keep in mind that many women with BV as defined by NS are totally asymptomatic and for this reason some investigators in the field believe this represents a normal variant of the vaginal microbiota [11]. Nevertheless, the microbiota associated with BV as defined by GS pattern is associated with a number of serious medical sequelae including preterm delivery [12], [13]. A reduction in the concentration of *Lactobacillus* species leads to an increase in vaginal pH and a deterioration in immune response to sexually transmitted viral infections including HIV [14]. Although the natural history of the microbial communities associated with BV is not yet fully understood [15], several studies suggest that the condition can be sexually transmitted [16], [17] and that *Gardnerella vaginalis* may be the etiologic agent [17]. In contrast to the latter assertion, *G. vaginalis* is also commonly detected in vaginal samples of women with GS-defined normal vaginal microbiota, albeit, at significantly lower concentrations than in GS-defined BV [18], [19], [20].

Phenotypic and genomic analyses of *G. vaginalis* isolates suggest that, in addition to low concentration, the conflicting observation of the presence of this species in both normal (or asymptomatic) and BV (or symptomatic) women may be rationalized by the existence of different strains of *G. vaginalis*, i.e. avirulent commensal strains colonize normal women while more-virulent strains may be infecting BV patients. This idea is supported by phenotypic analyses that show biofilm formation is a virulence trait of *G. vaginalis* isolates and the ability to form biofilms is associated with BV [21]. In addition, a recent genomic study showed that a *G. vaginalis* isolate from a GS-defined BV patient, differed from an isolate from a GS-defined normal patient by having the capacity to form tightly adherent biofilms on vaginal epithelial cells [22]. Genomic analysis of three *G. vaginalis* strains, two isolated from GS-defined BV patients and one from a GS-defined normal patient, showed that the GS-defined BV-associated strains produce proteins that are not found in the strain isolated from the GS-defined normal patient [23]. Moreover, another study of three *G. vaginalis* isolates revealed that two of the three isolates were able to produce sialidase, an enzyme associated with adverse pregnancy outcome in GS-defined BV patients [24], [25].

Piot et al. introduced a way to define *G. vaginalis* biotypes using enzymatic assays for lipase, hippurate hydrolysis and β-galactosidase activities [26], and defined eight biotypes. However, since eight (2^3^) is the maximum number of different types that can be defined using such an approach, the results may have reached that number not because the biotyping scheme is able to distinguish among all potential strains, but because the approach reached it's limit by finding all eight possible patterns of expression among the isolates. Hence, one cannot tell from these results whether in fact there may be more biotypes. Regardless, given the great diversity in human-host microbial communities, a new approach that has the potential to distinguish more biotypes may indeed reveal more types of *G. vaginalis*.

We explored the diversity and sexual transmissibility of *G. vaginalis* by examining the sequence variation and distribution of 65,710 *G. vaginalis* 16S rRNA pyrosequencing reads that were PCR-amplified from vaginal samples of 35 GS-defined BV, 5 GS-defined intermediate and 8 GS-defined normal women and from penile skin and urethral samples obtained from their male sexual partners. To identify high quality *G. vaginalis* sequences in our pyrosequencing libraries, and to minimize variation due to pyrosequencing errors, we performed a stringent search against a local database of 3 unique, full-length *G. vaginalis* 16S rRNA gene sequences acquired from the Ribosomal Database Project. We used a Shannon entropy-based approach to identify nucleotide positions that exhibit a high level of variation, and concatenated these nucleotides to define a set of 46 “*oligotypes”*. We examined patterns in the distribution and relative abundance of these oligotypes within individual couples, as well as across genders, anatomical sampling sites, and GS-defined BV and normal microbiota.

## Materials and Methods

### Ethics statement

All patients enrolled in this study signed written informed consent to their participation. The study protocol and consent form was approved by the LSU Health Sciences Center Institutional Review Board.

### Sample collection and clinical measurements

53 monogamous heterosexual couples were included in this study. The couples were recruited at the New Orleans STD clinic. From these 53 couples, we obtained 157 DNA samples (2 males did not provide urethral swabs). All subjects were at least 18 years old with no history of antibiotic use in the past 28 days, and couples presented together for evaluation. A vaginal swab was collected from each woman for DNA extraction and pyrosequencing analysis of bacterial composition. A separate vaginal swab sample was collected and characterized by GS NS [10]. The samples were designated “normal” (NS = 0–3), “intermediate” (NS = 4–6) or “BV” (NS = 7–10). Two urethral swabs and two penile skin swabs were collected from males. For penile skin samples, two sterile Copan flocked swabs were used. One was rolled with firm pressure around the circumference of the coronal sulcus and over the surface of the glans penis. The second one was rolled with firm pressure all over the penile shaft. Urethral swabs were collected by inserting a sterile swab into the urethral meatus and rotating back and forth for 2–3 seconds. The first urethral swab was rolled on a slide and stained with a modified methylene blue stain to evaluate for the presence of urethritis. The penile skin and second urethral swabs were immediately placed in individual sterile tubes containing 3 ml of DNA preservative (GeneLock^TM^, Sierra Molecular Corp., Sonora, CA).

### Molecular methods

Extraction of DNA from swab samples was performed using commercial kits according to the manufacturer's instructions. An initial bacterial cell lysis step using lysozyme (20 mg/ml at 37°C for 1 hour) was included (QIAamp DNA micro kit for male, QIAamp DNA mini kit for female samples, Qiagen Inc., Valencia, CA). DNA obtained from the coronal sulcus and penile shaft swabs was combined for the analyses of bacterial composition of penile skin. Bacterial tag-encoded FLX amplicon pyrosequencing (bTEFAP) was performed by the Research and Testing Laboratory (Lubbock, TX) using broad-range PCR-amplification of the approximately 570bp long V4 -V6 region of the 16S rRNA gene with primers 530F: GTGCCAGCMGCNGCGG and 1100R: GGGTTNCGNTCGTTG. Due to the difficulty extracting DNA from penile skin and urethra samples, amount of DNA per PCR reaction ranged from 1ng to 25ng (25ng per vaginal sample, 10ng per urethra sample, 1ng to 5ng per penile skin sample).

### Pyrosequencing analysis and extracting *G. vaginalis* sequences

Pyrosequencing analysis of all samples generated a total of 1,106,703 reads from 157 DNA samples. Of the total reads, 14.48% were discarded during the quality control step; 112,537 of these were short sequences (<200bp), 44,925 had one or more ambiguous bases, 1,022 had a mean quality score below Q25, and 1,838 had a single homopolymer region longer than 6 nucleotides. The average length of resulting 946,381 sequences that passed quality control was 481 nucleotides, with a standard deviation of 71, and the average number of sequences per sample was 6,257 with a standard deviation of 3,518. In order to identify and segregate the *G. vaginalis* reads from the rest of the sequences in the pyrosequencing library, we created a local database using three unique full-length *G. vaginalis* 16S rRNA gene sequences, acquired from the Ribosomal Database Project (the GenBank accession numbers: EF194095; CP001849; HQ641662). All 946,381 sequences were queried against this local search database using USEARCH [27] (version 4.2.66, with *e* value of 1e-30). Sequences that were ≥99% homologous to at least one of the *G. vaginalis* sequences in the local search database with a minimum alignment length of 480bp were retained for further analysis. The resulting *G. vaginalis* sequences were aligned to the GreenGenes [28] gold standard 16S rRNA gene sequence template for *G. vaginalis* using MUSCLE [29] and the ends were trimmed in order to reduce the variation in length. The minimum alignment length required for sequences to be retained as *G. vaginalis* during the database search was very close to the length of the sequence itself, hence we were unlikely to have chimeric sequences in our dataset. Nonetheless, we used UCHIME [30] to search for chimeras within the library in *de novo* mode, and no chimeric sequences were detected. A total of 65,710 quality-controlled and chimera-checked *G. vaginalis* sequences with the average nucleotide length of 481bp and a standard deviation of 1 nucleotide were used in further analyses. Some samples did not yield any *G. vaginalis* sequences that met the criteria described above, and these samples were excluded from the analysis. Table 1 shows the number of samples in the original pyrosequencing library compared to the number of samples per environment that had at least one *G. vaginalis* sequence meeting the criteria described above. The total number of sequences per sample in each original pyrosequencing library and the number of *G. vaginalis* sequences in the each library is shown in Table S1.

**Table 1:** Pyrosequencing analysis and USEARCH results summary.

Sample	Gram stain classification	# samples in the original pyrosequencing library	# samples after USEARCH search for *G. vaginalis*	Average # of *G. vaginalis* sequences per category
Vagina	BV	36	35	857
Vagina	Intermediate	5	5	525
Vagina	Normal	12	8	19
Penile skin	BV	36	30	209
Penile skin	Intermediate	5	5	25
Penile skin	Normal	12	6	26
Urethra	BV	36	29	660
Urethra	Intermediate	3	3	838
Urethra	Normal	12	9	473

Number of samples in the original pyrosequencing library compared to the number of samples per environment that had at least one high quality *G. vaginalis* 16S rRNA gene tag sequence that was ≥99% identical to one of 3 unique, full-length *G. vaginalis* 16S rRNA sequences obtained from the RDP.

### Identifying variable nucleotide positions and generating oligotypes

**Figure 1:**
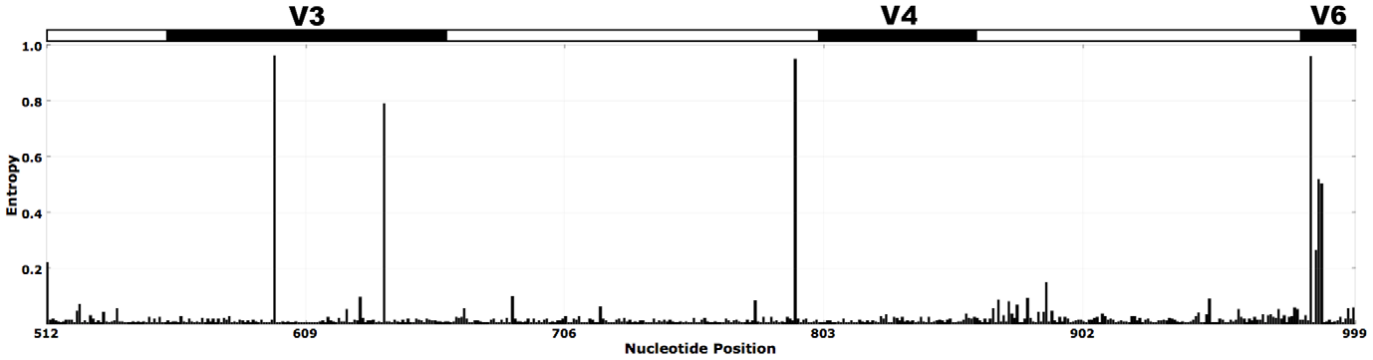
Shannon entropy analysis per column for 65,710 aligned *G. vaginalis* sequences. Peaks in entropy indicate nucleotide variation at given locations. While the X-axis of the figure indicates the location of the given column in the full length *G. vaginalis* 16S rRNA gene sequence, bar at the top superimposes the approximate locations of hyper-variable regions V4 (557–662), V5 (800–861) and V6 (981–1027) on *G. vaginalis* sequences that were used in this study.

We have implemented a program in Python (available from http://python.org) to perform Shannon entropy analysis on aligned *G. vaginalis* sequences to quantify the uncertainty due to nucleotide variation along the columns of aligned sequences in order to identify highly variable nucleotide positions. With this method we identified eight nucleotide positions that showed high variation in the V4–V6 region of *G. vaginalis* 16S rRNA gene (Figure 1). The variable locations that emerged from this analysis coincided with 511^st^, 612^th^, 661^st^, 835^th^, 988^th^, 989^th^, 990^th^ and 991^st^ nucleotide positions of the 16S rRNA gene from the genome sequence of *G. vaginalis* strain 409-05 (GenBank accession number: CP001849). None of these positions were associated with homopolymer regions, and nucleotide variations at these locations were also observed in some of the full-length *G. vaginalis* 16S rRNA gene sequences found in the RDP database. For each sequence in the tag library, we retained nucleotides only from those highly variable nucleotide positions and merged them into eight nucleotide oligomers, and used these oligomers to label individual *G. vaginalis* ‘*oligotypes’*. To reduce the probability of including an oligotype containing a nucleotide that may have been randomly generated by a sequencing error, we used only those oligotypes that were present in at least two samples. The resulting 46 oligotypes were used to generate *G. vaginalis* oligotype profiles for individual samples.

### Analyzing correlations among oligotype profiles

We used SciPy, an open-source scientific computation library (available from http://scipy.org/) for Python programming language, to compute Pearson correlation coefficients and p-values in order to explore linear relationships between sexual partners based on their *G. vaginalis* oligotype profiles. Pearson correlations were computed over the feature vectors that were constructed based on the percent abundance of oligotypes for every sample. The number of reads representing each oligotype was tallied for each sample to generate a 46-dimensional feature vector where each component of the vector reflected the percent abundance of the corresponding oligotype within the given sample. Pearson correlation analysis results are listed in Table 2.

**Table 2:** Pearson correlation (r) between sexual partners based on oligotype profiles.

Couple	Female Patient	Sex Partner's Penile Skin Sample			Sex Partner's Urethra Sample		
	# sequences	# sequences	r	p	# sequences	r	p
BV 01	1396	3	0.359	0.014	0	-	-
BV 02	1658	0	-	-	8	0.017	0.910
BV 03	1188	4	0.014	0.923	214	0.171	0.253
BV 05	416	10	**0.973**	< 0.001	2346	**0.962**	< 0.001
BV 06	268	39	0.855	< 0.001	0	-	-
BV 07	453	2	**0.983**	< 0.001	533	0.657	< 0.001
BV 08	1316	0	-	-	183	< 0.1	0.504
BV 09	689	49	**0.987**	< 0.001	209	0.134	0.371
BV 10	1046	2	0.030	0.839	4	0.030	0.839
BV 11	595	470	**0.970**	< 0.001	889	0.194	0.194
BV 12	166	67	0.679	< 0.001	503	0.642	< 0.001
BV 13	1941	68	0.864	< 0.001	245	0.830	< 0.001
BV 14	305	400	**0.981**	< 0.001	1045	0.819	< 0.001
BV 15	853	52	**0.921**	< 0.001	242	**0.992**	< 0.001
BV 17	911	32	0.838	< 0.001	100	**0.915**	< 0.001
BV 18	1323	17	**0.773**	< 0.001	0	-	-
BV 19	125	0	-	-	14	0.050	0.741
BV 20	542	35	**0.998**	< 0.001	363	**0.988**	< 0.001
BV 21	455	5	0.075	0.619	126	**0.995**	< 0.001
BV 22	2702	3646	**0.998**	< 0.001	179	0.820	< 0.001
BV 23	331	5	0.352	0.016	20	**0.995**	< 0.001
BV 24	678	1	0.675	< 0.001	50	0.675	< 0.001
BV 25	560	23	0.466	0.001	1383	0.467	0.001
BV 26	885	26	0.225	0.132	562	**0.983**	< 0.001
BV 27	1292	6	**0.976**	< 0.001	1323	**0.995**	< 0.001
BV 28	322	71	**0.951**	< 0.001	917	0.04	0.759
BV 29	856	1306	**0.959**	< 0.001	4738	0.519	0.001
BV 30	1382	4	0.107	0.476	0	-	-
BV 31	816	2	0.043	0.774	18	0.105	0.486
BV 32	185	4	0.744	< 0.001	59	0.837	< 0.001
BV 33	357	2	0.606	< 0.001	2353	0.793	< 0.001
BV 34	1219	17	**0.993**	< 0.001	8	0.265	0.074
BV 35	918	17	**0.995**	< 0.001	538	**0.979**	< 0.001
IN 01	647	50	0.344	0.019	0	-	-
IN 02	2	56	0.004	0.978	173	0.007	0.958
IN 03	1202	11	**0.990**	< 0.001	0	-	-
IN 04	274	5	**0.997**	< 0.001	680	**0.979**	< 0.001
IN 05	502	4	**0.961**	< 0.001	1661	0.185	0.217
N 03	2	129	0.557	< 0.001	2116	0.510	< 0.001
N 05	11	3	0.022	0.883	1	0.022	0.883
N 06	34	6	**0.918**	< 0.001	958	**0.980**	< 0.001
N 08	6	0	-	-	748	**0.998**	< 0.001
N 10	92	3	**0.931**	< 0.001	0	-	-
N 11	5	0	-	-	5	0.828	< 0.001

The oligotype profile of every female patient's vaginal sample compared to the oligotype profile of the urethral and penile skin samples of her sexual partner. The male partners of four women did not yield any *G. vaginalis* sequences, hence are not included in this table.

### Phylogenetic analysis of oligotypes and UniFrac clustering

**Figure 2:**
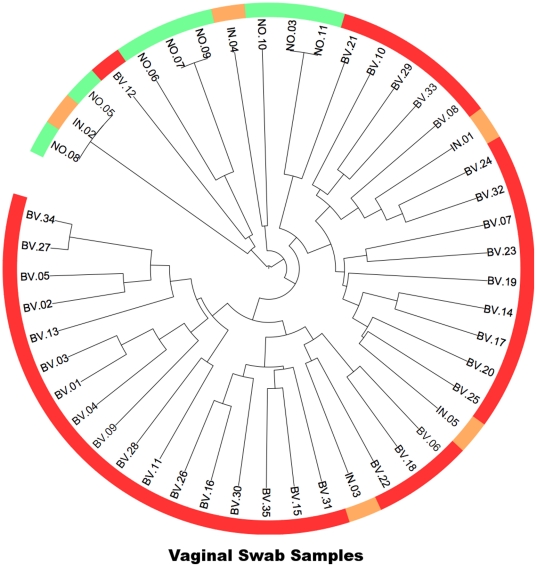
Hierarchical clustering results of vaginal swab samples. Samples were clustered (clustering significance: p<0.001, UniFrac significance: p = 0.016) based on the UniFrac distance metric. Red, orange and green colors indicate samples from BV, intermediate and normal Nugent categories, respectively.

Phylogenetic relationships among the oligotypes were assessed with Bayesian inference using MrBayes (version 3.1.2, http://mrbayes.sourceforge.net/) [31], [32]. Analysis was initiated with random starting trees with representative sequences for each oligotype, and posterior probabilities were determined from two independent runs of one million generations of Markov chain Monte Carlo simulations, from which tree topologies were sampled every 100 generations. After discarding the first 25% of resulting trees, a consensus phylogenetic tree of oligotypes was estimated from remaining generations (Figure S1). The resulting tree was used as a common phylogeny to perform UniFrac analysis [33]. Hierarchical clustering of oligotypes in vaginal (Figure 2), and penile skin and urethra samples (Figure S2) was performed based on distance matrices generated by the unweighted UniFrac analysis. Tree topology of the phylogenetic analysis and clustering results were visualized using the Interactive Tree of Life [34].

## Results

**Figure 3:**
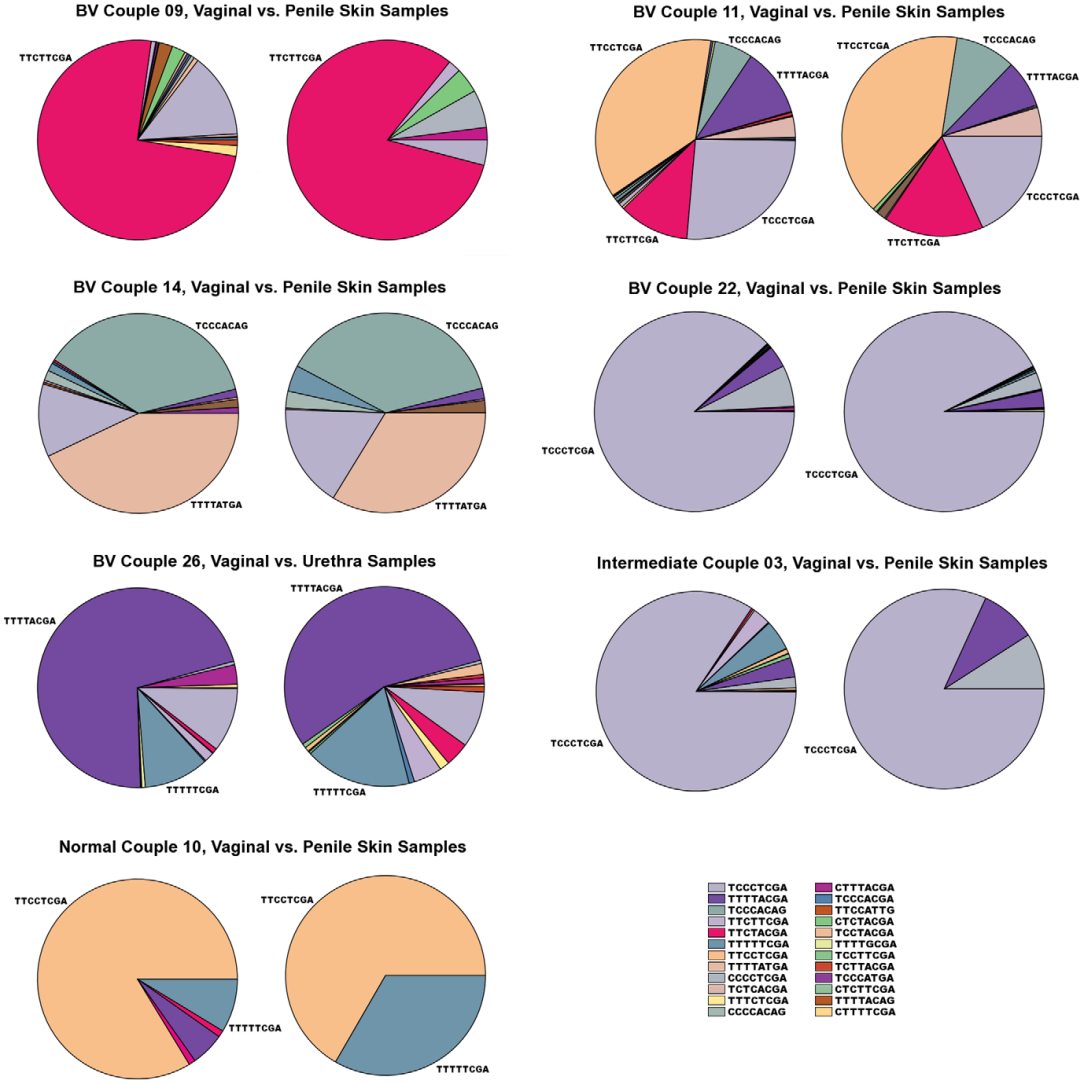
Oligotype profiles in various female patients and their sexual partners. Different colors in the pie charts correspond to different oligotypes. In every set, the pie chart on the left represents the sample collected from the female patient.

The generation of oligotype profiles by merging nucleotides from variable locations of *G. vaginalis* tag sequences that are revealed by Shannon entropy analysis (Figure 1) made it possible to compare samples to each other based on their *G. vaginalis* oligotype compositions. This analysis showed extensive diversity within *G. vaginalis* sequences from different samples, as well as significant correlations between the oligotype profiles of many couples. The composition of *G. vaginalis* oligotypes in vaginal samples of 24 of 44 women, whose partners also harbored at least one *G. vaginalis* sequence, were significantly correlated (r≥0.9, p<0.001) with either the penile skin, or urethral, or both samples from their sexual partners (Table 2). Significant correlation in *G. vaginalis* oligotypes was observed between vaginal and penile skin samples in 19 couples, while for vaginal and urethral samples of only 12 couples had correlation values above 0.9. In 8 couples, there was reduced, but nonetheless high degree of correlation (r≥0.5, p<0.001) between the vaginal and either the penile skin or the urethral samples. In 12 couple no correlation was found between partners (r<0.5). Figure 3 illustrates seven couples whose *G. vaginalis* compositions are highly correlated (see Figure S3 for stacked bar chart comparison of all samples). Correlation levels between partners did not appear to vary significantly by GS classification, although, the total number of couples in the intermediate and normal categories is small and the total number of *G. vaginalis* sequences in normal couples is low (Table 1).

The oligotype TCCCTCGA was the most abundant overall and it was observed in most of the samples (Table S2). It was the dominant oligotype of 24 of 48 vaginal samples. The TCCCACAG oligotype was the dominant oligotype in 10 vaginal samples. While the TTTTACGA, TTCTACGA and TTCCTCGA were dominant in 3 vaginal samples each, oligotypes TTTTATGA, TTCTTCGA and TCTCACGA were dominant in one vaginal sample each. A complete list of oligotype distribution across genders, anatomical sampling sites, and GS-defined BV and GS-defined normal microbiota is given in Table S2.

UniFrac [33], [35] is a computational method used to compare microbial samples to each other based on their composition with respect to a common phylogeny. After computing a phylogenetic tree for oligotypes using Bayesian inference, we used UniFrac to quantify similarities between samples based on their oligotype composition. Hierarchical clustering analysis on the UniFrac distance matrix of GS-defined BV and GS-defined normal vaginal samples grouped separately based on GS definition (Figure 2). Analogous comparison of urethral and penile skin samples from male partners of women with GS-defined BV or GS-defined normal vaginal flora did not show a similar separation (Figure S2).

## Discussion

It is well known that bacterial species with identical 16S rRNA genes can represent different ecotypes with differences in virulence properties and other phenotypic traits [36]. In this sense, the sensitivity of the 16S rRNA gene is limited, but it is specific; it has been shown that even one nucleotide difference at the level of the 16S rRNA gene may be an indicator of an ecologically distinct strain [37]. Moreover, there is a correlation between 16S rRNA gene divergence and the overall gene content [38], and the evolutionary distances of 16S rRNA genes can be used to discern genomic differences between species even with short pyrosequencing tag reads [39].

With the availability and affordability of massively parallel high-throughput sequencing technologies it is now possible to collect vast amounts of sequence data that cover a great deal of bacterial diversity within an environmental sample without the need for cultivation [40]. However, due to the nature of pyrosequencing, sequences contain biologically irrelevant random sequencing errors, rendering them imprecise and noisy for inferring diversity at very low levels of taxonomy with high confidence. For instance, the two 16S rRNA genes of two *G. vaginalis* strains used in a genomic comparison study [22] differed by only 6 nucleotides at the 16S rRNA gene level, which was equivalent to 0.38% variation. Nevertheless, these two strains with very low level of variation at the 16S rRNA gene level were significantly different from each other in respect to their whole genomes. However, 0.38% variation is lower than the expected 1% random error rate of pyrosequencing [41], and very close to the expected 0.25% random error rate of pyrosequencing reads after stringent quality filtering [42]. As a result of this, such low levels of variation are beyond the capacity of commonly available computational methods to separate these variants confidently, resulting in strains that are similar at the 16S rRNA gene level to receive the same taxonomical assignment, or to be collected in one OTU group. Similarly, variation across the sequences we analyzed in this study ranged from 0.2% to 1.66% over the 481 nucleotide-long pyrosequencing reads obtained from the V4–V6 region of the 16S rRNA gene. Therefore, due to the very high similarity among sequences, all would have been considered *G. vaginalis*, or clustered in one 3% OTU group. In spite of this, we observed a remarkable amount of *G. vaginalis* diversity, and were able to detect a high degree of correlation between oligotype profiles of many sexual partnerships (see Figure 3).

This relatively large scale study of variation in *G. vaginalis* 16S rRNA gene sequences supports previous cultivation-based studies that suggest *G. vaginalis* is sexually transmissible and that male and female partners share similar *G. vaginalis* strains [17], [43]. Moreover, results of this study show that the usual approaches used to analyze 454 pyrosequencing data derived from human genitourinary tract samples miss important diversity that may be ecologically, clinically and/or epidemiologically significant.

The UniFrac analysis results appear to suggest that there may be a unique, closely related group of *G. vaginalis* oligotypes found among GS-defined normal and some GS-defined intermediate women. However, the relatively limited number of GS-defined normal and GS-defined intermediate women included in this study, require these results to be corroborated by additional studies. Nonetheless, results presented here suggest that the oligotyping approach could be used to identify and separate very similar strains at 16S rRNA gene level from high-throughput sequencing data, and explore whether there are specialized types for different ecological niches. Preliminary analysis of *Megasphaera* spp. has also revealed numerous oligotype distribution profiles among women with GS BV (results not shown), suggesting that applying the method described here to other species that are commonly found in the genitourinary microbiota could yield important new insights. Additionally, consideration should be given to oligotype analyses of other phylogenetically informative genes, such as *recA* [44], [45], in order to explore to which extent the oligotypes at the 16S rRNA gene level are able to predict genomic variation.

In summary, our study describes a novel method for revealing concealed diversity at a very low level of taxonomy by utilizing Shannon entropy to amplify weak signals of subtle but reproducible nucleotide variation within high-throughput sequencing reads. This oligotyping approach can be applied to existing sequence libraries to explore diversity at an ecologically meaningful level and investigate potential ecotypes and their diversity hidden within conventionally defined species.

## Supporting Information

Figure S1 Phylogenetic distribution of 46 oligotypes. Bars and numbers next to oligotypes indicate how many samples they were present at least once in all samples.
(TIFF)

Figure S2 Hierarchical clustering results of samples from male patients. Penile skin (clustering significance: p = 0.011, UniFrac significance: p = 0.001) and urethra (clustering significance: p<0.001, UniFrac significance: p = 0.077) samples were clustered based on the UniFrac distance metric. Red, orange and green colors indicate samples that are sexual partners of GS-defined BV, GS-defined intermediate and GS-defined normal female patients, respectively.
(TIFF)

Figure S3 Stacked bar representation of oligotype profiles among couples. While VS labeled bars represent female patients oligotype profile, for every couple MP (penile skin sample) or MU (urethra sample) bars represent male sexual partners oligotype profile. For the sake of compactness, only the more similar sample to vaginal sample from male partner were used when both MP and MU samples were available for a given couple.
(TIFF)

Table S1 Number of sequences in each sample. Number of sequences per sample in the original pyrosequencing library versus the number of sequences that were 99% or more similar to one of the full length *G. vaginalis* sequences in the local search database with minimum alignment length of 480bp.
(DOC)

Table S2 Oligotype distribution among sample groups. Every column in this table shows the number of samples in a group in which the given oligotype was observed at least once. The total number of vaginal swab (VS), penile skin (PS), and urethral (U) samples are shown in parentheses in all three groups of Gram Stain (GS) BV, GS Intermediate and GS Normal.
(DOC)
