# Supplementary figures and images for: Exploring the Diversity of Gardnerella vaginalis in the Genitourinary Tract Microbiota of Monogamous Couples Through Subtle Nucleotide Variation
Source: PLoS One. 2011 Oct 25;6(10):e26732. doi: 10.1371/journal.pone.0026732 (PMC3201972; doi:10.1371/journal.pone.0026732)

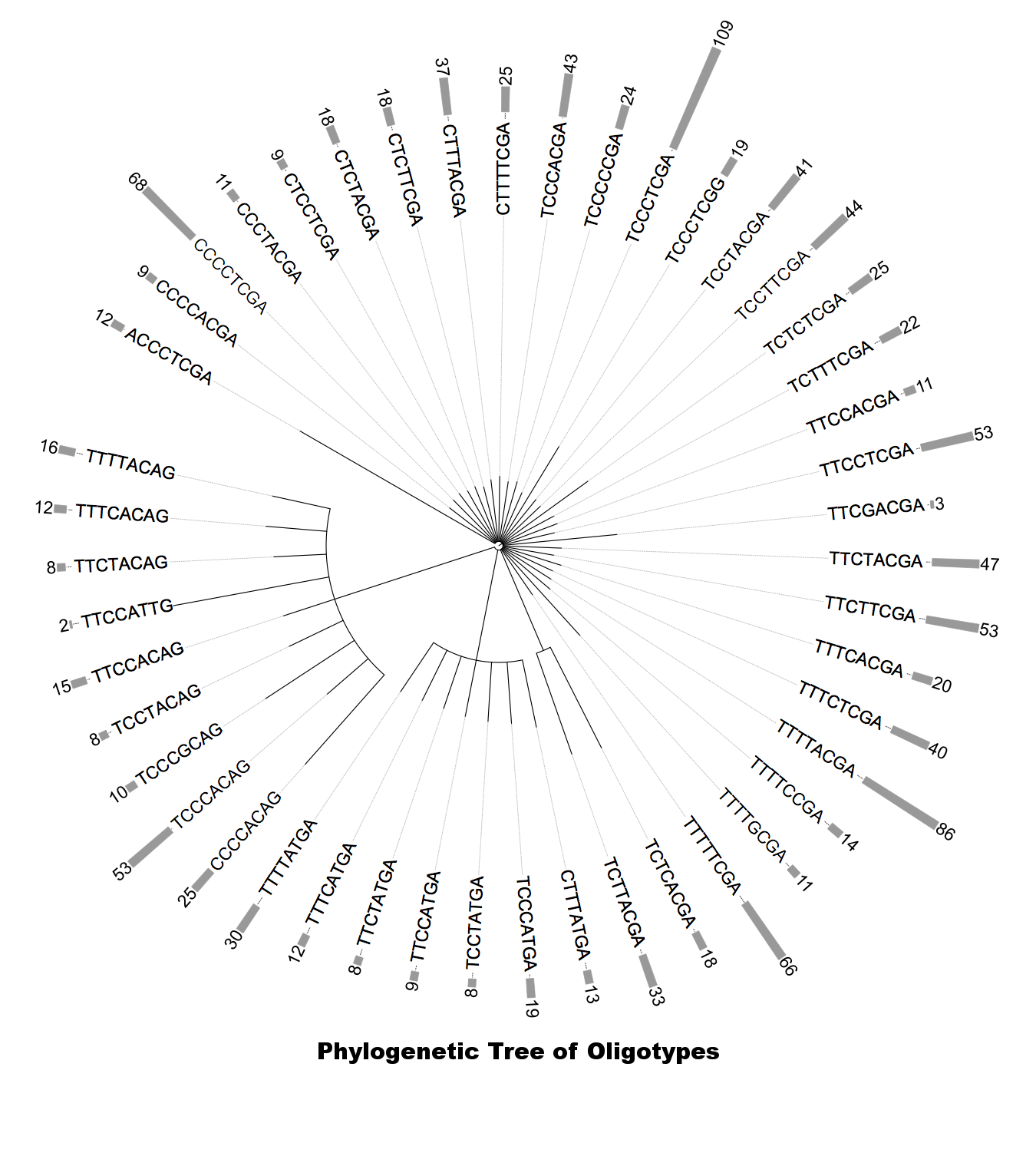

Supplement: Figure S1 — Phylogenetic distribution of 46 oligotypes. Bars and numbers next to oligotypes indicate how many samples they were present at least once in all samples. (TIFF) [file pone.0026732.s001.tif]

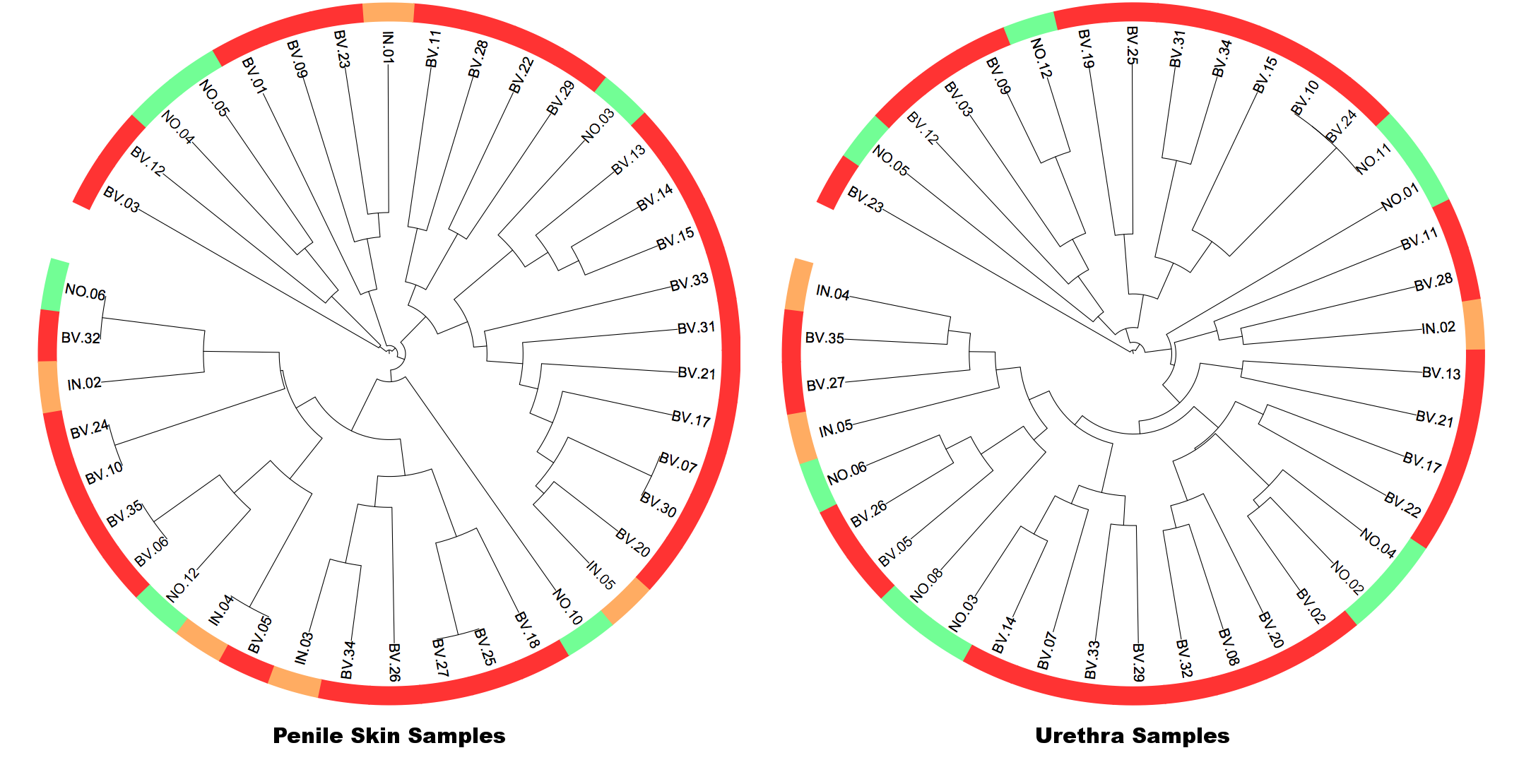

Supplement: Figure S2 — Hierarchical clustering results of samples from male patients. Penile skin (clustering significance: p = 0.011, UniFrac significance: p = 0.001) and urethra (clustering significance: p<0.001, UniFrac significance: p = 0.077) samples were clustered based on the UniFrac distance metric. Red, orange and green colors indicate samples that are sexual partners of GS-defined BV, GS-defined intermediate and GS-defined normal female patients, respectively. (TIFF) [file pone.0026732.s002.tif]

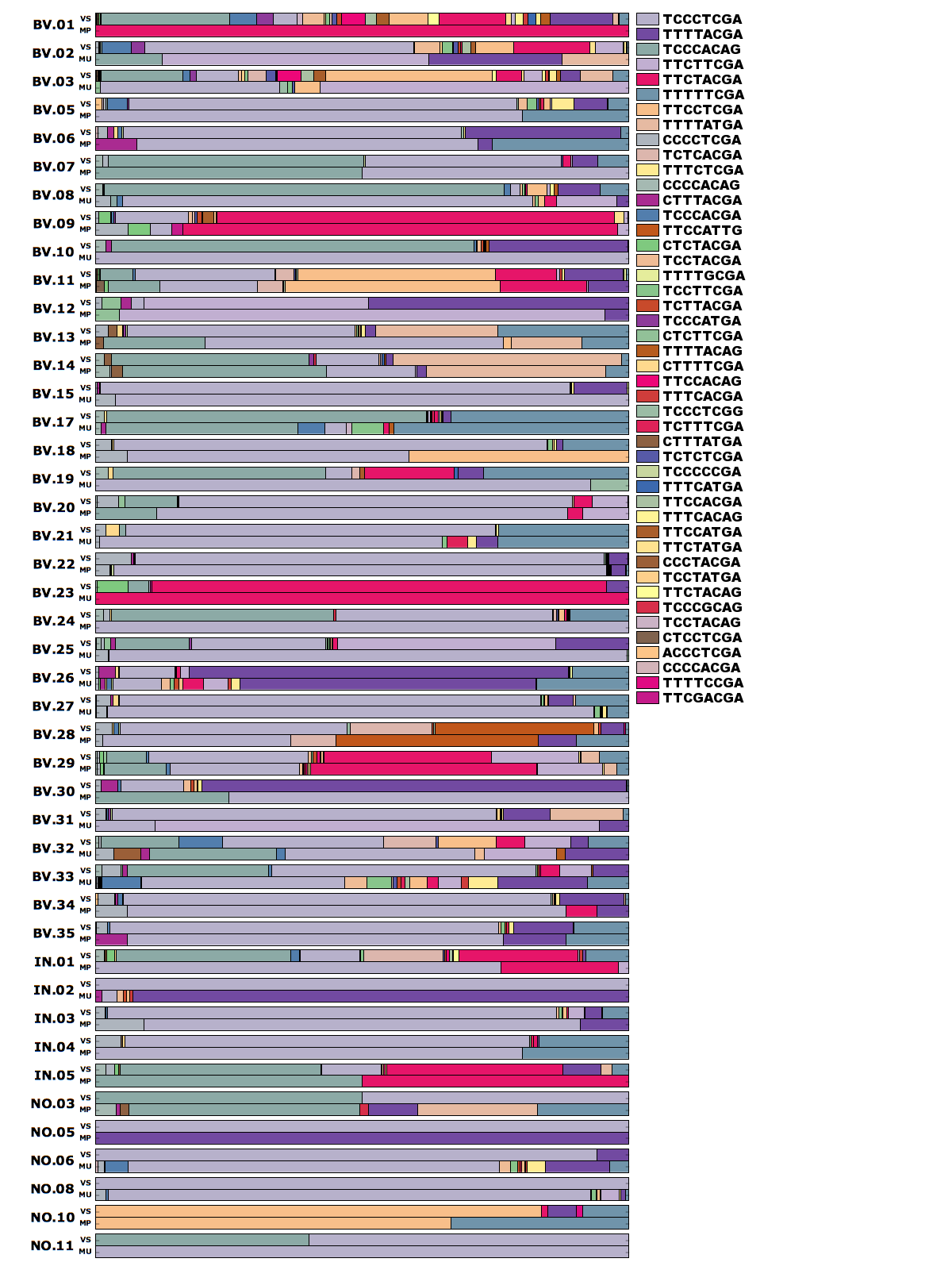

Supplement: Figure S3 — Stacked bar representation of oligotype profiles among couples. While VS labeled bars represent female patients oligotype profile, for every couple MP (penile skin sample) or MU (urethra sample) bars represent male sexual partners oligotype profile. For the sake of compactness, only the more similar sample to vaginal sample from male partner were used when both MP and MU samples were available for a given couple. (TIFF) [file pone.0026732.s003.tif]
