# Supplementary material for: Exploring the Diversity of Gardnerella vaginalis in the Genitourinary Tract Microbiota of Monogamous Couples Through Subtle Nucleotide Variation
Source: PLoS One. 2011 Oct 25;6(10):e26732. doi: 10.1371/journal.pone.0026732 (PMC3201972; doi:10.1371/journal.pone.0026732)
Supplement: Table S1 — Number of sequences in each sample. Number of sequences per sample in the original pyrosequencing library versus the number of sequences that were 99% or more similar to one of the full length G. vaginalis sequences in the local search database with minimum alignment length of 480bp. (DOC) [file pone.0026732.s004.doc]

| Couple | Female Patient | | Sex Partner's Penile Skin Sample | | Sex Partner's Urethra Sample | |
| --- | --- | --- | --- | --- | --- | --- |
|  | # of all sequences | # of *G. vaginalis* sequences | # of all sequences | # of *G. vaginalis* sequences | # of all sequences | # of *G. vaginalis* sequences |
| BV 01 | 6424 | 1396 | 3947 | 3 | 0 | 0 |
| BV 02 | 4377 | 1658 | 6053 | 0 | 3924 | 8 |
| BV 03 | 7772 | 1188 | 10038 | 4 | 3319 | 214 |
| BV 05 | 2022 | 416 | 6326 | 10 | 5367 | 2346 |
| BV 06 | 9253 | 268 | 1175 | 39 | 0 | 0 |
| BV 07 | 9254 | 453 | 742 | 2 | 6005 | 533 |
| BV 08 | 9295 | 1316 | 0 | 0 | 4907 | 183 |
| BV 09 | 7497 | 689 | 434 | 49 | 3264 | 209 |
| BV 10 | 8088 | 1046 | 9237 | 2 | 4125 | 4 |
| BV 11 | 7052 | 595 | 7578 | 470 | 6261 | 889 |
| BV 12 | 5907 | 166 | 8676 | 67 | 7190 | 503 |
| BV 13 | 4118 | 1941 | 9317 | 68 | 4767 | 245 |
| BV 14 | 5293 | 305 | 18796 | 400 | 6858 | 1045 |
| BV 15 | 8908 | 853 | 2092 | 52 | 6392 | 242 |
| BV 17 | 6216 | 911 | 8159 | 32 | 7968 | 100 |
| BV 18 | 4303 | 1323 | 9423 | 17 | 8481 | 0 |
| BV 19 | 7404 | 125 | 7502 | 0 | 4580 | 14 |
| BV 20 | 5430 | 542 | 7536 | 35 | 2685 | 363 |
| BV 21 | 6538 | 455 | 5455 | 5 | 1424 | 126 |
| BV 22 | 6720 | 2702 | 16744 | 3646 | 2444 | 179 |
| BV 23 | 8631 | 331 | 14849 | 5 | 2069 | 20 |
| BV 24 | 9482 | 678 | 5410 | 1 | 4464 | 50 |
| BV 25 | 5308 | 560 | 5978 | 23 | 3371 | 1383 |
| BV 26 | 3563 | 885 | 3221 | 26 | 9266 | 562 |
| BV 27 | 5174 | 1292 | 1331 | 6 | 3387 | 1323 |
| BV 28 | 5718 | 322 | 6557 | 71 | 3437 | 917 |
| BV 29 | 5013 | 856 | 18249 | 1306 | 8399 | 4738 |
| BV 30 | 6197 | 1382 | 13662 | 4 | 0 | 0 |
| BV 31 | 3466 | 816 | 1594 | 2 | 5942 | 18 |
| BV 32 | 5306 | 185 | 4296 | 4 | 6359 | 59 |
| BV 33 | 7776 | 357 | 1558 | 2 | 5240 | 2353 |
| BV 34 | 11720 | 1219 | 355 | 17 | 3470 | 8 |
| BV 35 | 9012 | 918 | 4866 | 17 | 2188 | 538 |
| IN 01 | 5257 | 647 | 10947 | 50 | 0 | 0 |
| IN 02 | 4484 | 2 | 7738 | 56 | 3913 | 173 |
| IN 03 | 6200 | 1202 | 4638 | 11 | 0 | 0 |
| IN 04 | 7162 | 274 | 13088 | 5 | 4641 | 680 |
| IN 05 | 4737 | 502 | 149 | 4 | 6100 | 1661 |
| N 03 | 4333 | 2 | 3565 | 129 | 7069 | 2116 |
| N 05 | 5849 | 11 | 6579 | 3 | 6530 | 1 |
| N 06 | 4287 | 34 | 8372 | 6 | 4169 | 958 |
| N 08 | 2465 | 6 | 0 | 0 | 2989 | 748 |
| N 10 | 7454 | 92 | 155 | 3 | 0 | 0 |
| N 11 | 5279 | 5 | 0 | 0 | 4465 | 5 |
