# Supplementary material for: Exploring the Diversity of Gardnerella vaginalis in the Genitourinary Tract Microbiota of Monogamous Couples Through Subtle Nucleotide Variation
Source: PLoS One. 2011 Oct 25;6(10):e26732. doi: 10.1371/journal.pone.0026732 (PMC3201972; doi:10.1371/journal.pone.0026732)
Supplement: Table S2 — Oligotype distribution among sample groups. Every column in this table shows the number of samples in a group in which the given oligotype was observed at least once. The total number of vaginal swab (VS), penile skin (PS), and urethral (U) samples are shown in parentheses in all three groups of Gram Stain (GS) BV, GS Intermediate and GS Normal. (DOC) [file pone.0026732.s005.doc]

|  | Gram stain BV | | | Gram stain Intermediate | | | Gram stain Normal | | |
| --- | --- | --- | --- | --- | --- | --- | --- | --- | --- |
| Oligotype | VS (35) | PS (30) | U (29) | VS (5) | PS (5) | U (3) | VS (8) | PS (6) | U (9) |
| TCCCTCGA | 33 | 25 | 25 | 5 | 4 | 3 | 5 | 2 | 7 |
| TTTTACGA | 32 | 13 | 18 | 4 | 2 | 3 | 4 | 5 | 5 |
| CCCCTCGA | 29 | 12 | 16 | 4 | 1 | 2 | 0 | 0 | 4 |
| TTTTTCGA | 27 | 11 | 12 | 4 | 1 | 1 | 1 | 3 | 6 |
| TCCCACGA | 25 | 2 | 9 | 2 | 0 | 2 | 0 | 0 | 3 |
| TCCCACAG | 22 | 11 | 10 | 2 | 1 | 1 | 2 | 1 | 3 |
| TTCTTCGA | 22 | 8 | 12 | 2 | 1 | 1 | 0 | 0 | 7 |
| TCCTACGA | 21 | 2 | 9 | 3 | 0 | 2 | 0 | 0 | 4 |
| TTCTACGA | 20 | 8 | 9 | 4 | 2 | 1 | 1 | 0 | 2 |
| TTCCTCGA | 20 | 5 | 16 | 3 | 0 | 3 | 1 | 1 | 4 |
| CTTTACGA | 20 | 3 | 6 | 1 | 0 | 3 | 0 | 2 | 2 |
| TTTCTCGA | 20 | 2 | 11 | 1 | 0 | 2 | 0 | 0 | 4 |
| TCTTACGA | 19 | 3 | 6 | 1 | 0 | 2 | 0 | 0 | 2 |
| TCCTTCGA | 18 | 2 | 15 | 3 | 0 | 2 | 0 | 0 | 4 |
| CCCCACAG | 14 | 3 | 4 | 2 | 0 | 0 | 0 | 1 | 1 |
| TCTCTCGA | 14 | 1 | 6 | 2 | 0 | 1 | 0 | 0 | 1 |
| TTTTATGA | 13 | 5 | 7 | 1 | 0 | 0 | 0 | 1 | 3 |
| CTTTTCGA | 13 | 1 | 6 | 3 | 0 | 0 | 0 | 0 | 2 |
| TCCCATGA | 12 | 0 | 5 | 1 | 0 | 0 | 0 | 0 | 1 |
| TCCCTCGG | 11 | 2 | 4 | 1 | 0 | 1 | 0 | 0 | 0 |
| TCTCACGA | 10 | 3 | 4 | 1 | 0 | 0 | 0 | 0 | 0 |
| TCCCCCGA | 9 | 2 | 9 | 0 | 0 | 2 | 0 | 0 | 2 |
| TCTTTCGA | 9 | 1 | 8 | 1 | 0 | 1 | 0 | 0 | 2 |
| TTTTACAG | 9 | 0 | 6 | 0 | 0 | 0 | 0 | 0 | 1 |
| CTCTACGA | 8 | 4 | 3 | 2 | 1 | 0 | 0 | 0 | 0 |
| CTCTTCGA | 8 | 3 | 5 | 1 | 0 | 0 | 0 | 0 | 1 |
| TTCCACAG | 8 | 2 | 2 | 2 | 0 | 0 | 0 | 0 | 1 |
| TTTTGCGA | 8 | 1 | 2 | 0 | 0 | 0 | 0 | 0 | 0 |
| TTTCACGA | 8 | 0 | 7 | 1 | 0 | 2 | 0 | 0 | 2 |
| TTTCATGA | 8 | 0 | 3 | 0 | 0 | 0 | 0 | 0 | 1 |
| TTCCACGA | 7 | 1 | 2 | 1 | 0 | 0 | 0 | 0 | 0 |
| TTTCACAG | 7 | 0 | 4 | 0 | 0 | 0 | 0 | 0 | 1 |
| TTTTCCGA | 6 | 1 | 4 | 1 | 0 | 0 | 1 | 0 | 1 |
| ACCCTCGA | 6 | 0 | 4 | 0 | 0 | 2 | 0 | 0 | 0 |
| TTCCATGA | 6 | 0 | 3 | 0 | 0 | 0 | 0 | 0 | 0 |
| TCCCGCAG | 6 | 0 | 1 | 1 | 0 | 0 | 0 | 1 | 1 |
| TTCTACAG | 5 | 1 | 1 | 1 | 0 | 0 | 0 | 0 | 0 |
| TTCTATGA | 5 | 1 | 1 | 0 | 0 | 0 | 0 | 0 | 1 |
| TCCTATGA | 5 | 0 | 2 | 0 | 0 | 0 | 0 | 0 | 1 |
| CCCCACGA | 5 | 0 | 2 | 0 | 0 | 0 | 0 | 0 | 2 |
| CCCTACGA | 4 | 1 | 5 | 1 | 0 | 0 | 0 | 0 | 0 |
| CTCCTCGA | 4 | 1 | 3 | 1 | 0 | 0 | 0 | 0 | 0 |
| CTTTATGA | 3 | 3 | 4 | 1 | 0 | 0 | 0 | 1 | 1 |
| TCCTACAG | 3 | 0 | 4 | 0 | 0 | 0 | 0 | 0 | 1 |
| TTCGACGA | 2 | 1 | 0 | 0 | 0 | 0 | 0 | 0 | 0 |
| TTCCATTG | 1 | 1 | 0 | 0 | 0 | 0 | 0 | 0 | 0 |
